# Outlines of Pathology and Practice of Medicine

**Published:** 1844-07

**Authors:** 


### Art. XVIII.

*Outlines of Pathology and Practice of Medicine.* By William Pulteney Alison, m.d. f.r.s.e., Professor of the Practice of Medicine in the University of Edinburgh, &c. &c.—*London and Edinburgh,* 1843-4. 8vo, pp. 736.

On the appearance of the first and second parts of this work, we expressed a very high opinion of its merits,—an opinion confirmed by perusing it in its now complete form, in which it demands a more detailed notice than it has hitherto received from us. The work is, in truth, unique among the publications of the present day; for it is full of profound and original views of the philosophy of medicine, while it presents to the student an admirably simple and accurate exposition of the nature and treatment of diseases.

Our author's mode of introducing his subject is very uncommon, yet strikingly just and apposite.

"As the object of physiology is to deliver the history and explanation of all the phenomena by which the living body is distinguished from the dead, so the object of pathology is to describe and explain all the phenomena by which the diseased states of the living body differ from the healthy; and we call all states of the living body diseased, in which there are such deviations from its natural condition, as cause suffering or inconvenience, or endanger life. A slight attention to this subject is sufficient to show, that there are many facts in regard to the operation of external causes on the human body, and the modes of diseased action assumed by its different organs, which could not possibly have been inferred from our knowledge of the structure and healthy action of parts, which are made known to us only by observation of the diseased conditions of the body themselves, and can only be properly generalized by an induction strictly confined to this department of knowledge. The fewer and more comprehensive these ultimate facts or laws in this department of nature, the more successful must the intluction be regarded; and some of them have been already so far ascertained, as to enable us to treat the subject in some measure *synthetically*. The simplest exemplification of these ultimate facts in pathology, is to be found in cases of sudden death, and in the action of violent injuries, and we therefore premise a short account of different fatal injuries, to discussions on the pathology of the different diseases." (pp. 1, 2.)

The first chapter, on "Cases of sudden or violent death," is a remarkable specimen of powerful and highly-condensed medical reasoning, and, though it occupies only twenty-nine pages, it contains matter that might be expanded into a most useful treatise on the analogy of injuries and diseases. This subject, though almost entirely neglected, abounds with the most valuable materials for pathological reasoning and practical inference. A principal cause of its having been so little cultivated resides, doubtless, in that pernicious schism between physic and surgery which so long impeded the progress of both those branches of medicine, but which the advanced state of the general science has now, happily, almost abolished. There are many points in the practice of surgery which might long ago have both suggested and elucidated important questions in the treatment of disease. How long, for example, had the treatment of ophthalmia and gonorrhea made surgeons familiar with the use of stimulants and tonics in inflammation of mucous membranes when passing from the acute to the chronic form, before a parallel procedure found its way into the practice of physic? And yet the principle here involved is one of considerable moment, and suggests, as a subject of practical inquiry, whether inflammation of every texture may not occasionally assume an aspect under which the use of stimulants has a direct tendency to subdue the morbid action? Stimulants have, indeed, been employed by physicians time immemorial in inflammatory cases, but this only with a view of sustaining the vital powers when sinking,—a use of stimulants differing widely in principle from that which we have just represented as deducible from the practice of surgery, and which has only very recently begun to enter into the speculations of the physician. Again, the experience of surgeons in injuries of the head might long ago have suggested to physicians the important fact of the representation of cerebral disease by vomiting and other abdominal symptoms,—a fact of frequent occurrence, though not perhaps even yet so familiar as it ought to be to many practitioners in medicine. It would be easy to multiply instances in support of our argument. Instead, however, of so doing, we will attempt to analyse the first chapter of Dr. Alison's work, which affords an excellent specimen of his style of reasoning.

All causes of sudden or violent death operate either by *directly depressing or suspending the vital actions of the organs of circulation,* or by *obstructing the arterialization of the blood,* and thence *arresting the circulation at the lungs.* The first effect, or *death by syncope,* may take place in two ways: 1, by the action of a cause depressing the vital powers by which the blood is moved—as a concussion; 2, by the sudden or gradual abstraction of the vital stimulus, i. e. the blood. The second effect, or arrest of the pulmonary circulation, may also take place in two ways: 1, by injury of the nervous system arresting respiration by causing insensibility, and producing *death by coma,* or death beginning at the brain; 2, by directly impeding the access of air to the lungs, producing *death by asphyxia,* or death beginning at the lungs. But though death be always occasioned by one or other of these causes, the same cause may act, under different circumstances, sometimes chiefly in one way, and sometimes in the other. To these principles we can ascribe the known effects of the following kinds of injury:

I. Injuries affecting the *nervous system* impair, more or less, sensation, volition, and the mental phenomena; but they become dangerous to life only as they affect the circulation, either directly, or through the medium of respiration.

1. The most violent injuries affecting the nervous system suddenly arrest, or greatly impair the motion of the blood in all parts of the body, i. e. they produce a state of syncope or faintness. Such effect on the heart's actions and on the capillary circulation may result from injury of any part of the brain or spinal cord, if it extend to large portions of the nervous matter. This state of syncope forms the most characteristic part of the first symptoms of general concussion of the brain, as distinguished from more partial compression of it; and we learn from many examples that when concussion is general over the whole system, it is frequently fatal in this way, without producing any visible disorganization. It is not quite certain, in cases of general concussion, that the fatal depression of the circulating system takes place through the medium of the nervous system, and some have supposed that such injuries are fatal by a direct effect on the circulation in the smaller arteries, checking it so completely as to oppose a resistance to the heart which it is unable to overcome; but since the effects of violent injuries, confined to the brain or spinal cord, are just similar to those of a general concussion,—since powerful mental causes, which certainly operate through the nervous system, have the same effect,—since partial, although violent, injury of other textures has no such effect,—and, lastly, since no such effect results from suddenly stopping the flow of blood in large arteries—it is highly probable that general concussion of the body does act on the vascular system through the intervention of the nervous, and that this is one of the facts included in the general physiological principle that the nervous system, although not necessarily concerned in the functions of organic life, is yet so connected with them, that, by certain changes in it, any of these functions may be variously altered or totally suspended. There is great variety as to the amount of injury which will produce this sedative effect on the circulation in different individuals of the human species, and as to the duration and termination of that effect, which sometimes abates quickly, and at others gradually increases till it is fatal. There is diversity also in the part of the circulating system chiefly affected by such injuries. In some cases the superficial circulation seems to be more reduced than in others where there is equal depression of the heart's action. In Chossat's experiments, the action of the heart was for some time little affected by certain injuries of the brain, which so checked the capillary circulation as to suspend secretion and calorification. Also, when the spinal cord, in the human subject, has been severely injured below the neck, the circulation in the capillaries has generally appeared, for some time, more affected than the action of the heart. There is likewise great variety in the effects of such injuries on the nervous system itself, which accompany or succeed the sedative effects on the circulation. Sometimes the coma is profound and long continued, sometimes it is slight and transient; sometimes there is much convulsion, at others little or none; in some cases the coma is succeeded by much headach, general or partial amentia or delirium, incessant nausea and vomiting, and, in other cases, by none of these. It is certain that all these various symptoms may exist independently of any perceptible change of structure in the nervous system. The sedative effect on the vascular, from injury of the nervous system, is seldom the immediate object of practice, because if it be not immediately fatal, it is usually followed by inflammation, against which the chief remedies must be directed: in a few cases, however, the danger from the first effects of the concussion may be averted by the cautious use of internal and external stimuli. It is important to observe that when injuries, affecting the nervous system, and through it the circulation, are not speedily fatal, but followed by inflammation and fever, the progress of these is frequently modified by the preceding or accompanying state of the system likewise consequent on the injuries, and the fever is apt to assume a typhoid, and the inflammation a gangrenous character. The relation of these facts to the true pathology of typhoid fever is still doubtful, but it is certain that in such states resulting from injury, the treatment adapted to typhoid fever is admissible, and sometimes decidedly beneficial.

2. Slighter, and especially more partial injuries of the brain, or upper part of the spinal cord, often produce death in a totally different way, namely by *coma*, the action of the heart remaining unimpaired, till the defect of respiration, occasioned most probably by loss of sensibility, at length brings the blood to a stand in the capillaries of the lungs, and this puts a final stop to the whole circulation. In characteristic cases of this kind the circulation, and even the animal heat, not only continue till the last breath is drawn, but may even survive respiration for a short time. The most common injuries of the nervous system which cause death by coma are those in which there is partial compression of the nervous matter; but as death happens in the same way from disorganizations of the brain, which do not necessarily involve compression of its substance, and from the action of certain poisons which appears to have no connexion with compression, it is incorrect to speak generally of coma and its accompanying symptoms as indications of pressure on the brain. There is great variety as to the duration and degree of the insensibility which precedes the failure of respiration in such cases, and also as to other affections which may precede or attend that insensibility, as head-ach, delirium, somnolency, spasms, palsy, dilated or contracted pupil, preternaturally slow, or frequent, or irregular pulse, &c. Even the respiration itself is variously affected, it being sometimes hurried and imperfect, in others preternaturally slow and deep, previously to its final suppression.

The two preceding modes in which injuries of the nervous system may cause death, though perfectly distinct in some cases, are combined in others, as in those injuries of the head where insensibility and faintness from concussion immediately succeed the accident, but quickly abate, and are followed after an interval, by insensibility and a full pulse, and death by coma, which may then be confidently ascribed to compression of the brain by effused blood or serum. There are various causes, physical and mental, which affect the nervous system nearly in the same manner as a concussion : such as the sudden diminution of pressure to which the brain and spinal cord have been previously subjected, as instanced in the removal of depressed portions of the cranium, coagula of blood, or accumulations of serum;—in the rupture of a large blood-vessel within the head, implying a sudden determination of pressure on a part of the nervous matters, and attended at first with insensibility and feebleness of the circulation, followed by recovery of sense, rising of the pulse, and lastly the gradual accession of fatal coma from increased effusion;—in the production of syncope by the erect posture during bloodletting, and the removal of the fluid of ascites in the absence of artificial compression;—and in the syncope or diminished force of the heart's action occasioned by rising suddenly after long stooping. Another cause acting in a manner analogous to concussion of the brain is a violent blow on the abdomen, and especially on the epigastrium; and the concussion from a severe and extensive wound of any part of the abdomen is usually fatal in the same rapid way, independently of hemorrhage, and before there is time for inflammation to be established. The effects of cold water drunk when the system is heated and exhausted are referrible to the same principle. These facts illustrate the more gradual depression of the power of the heart, which is observed in the earlier stages of inflammation, and other acute diseases of the abdominal viscera. Violent injuries of various other parts of the body, especially in persons of a weakly habit, and where the nervous system is in a state of unnatural excitability, as from the abuse of opium or alcohol, may also act in the manner of a concussion, either causing sudden death, or so depressing the actions of the vascular system, as to give a typhoid type to the fever, and gangrenous tendency to the inflammation, which are to result.

In such cases, Dr. Alison thinks, it can hardly be doubted that the violence, or peculiar nature, of the sensation which attends the injury is the intervening link through which the action of the heart is affected, and, accordingly, various violent or overpowering sensations, intense pain, or the sudden transition from pain to ease, and certain mental emotions or passions, as joy, grief, anger, fear, existing in their utmost intensity, affect the circulation just as a concussion does, and sometimes with a fatal result.*

II. The effect of very intense heat applied to a large surface of the body, as in an extensive burn, or to the whole body, as in a *coup de soleil,* is also quite similar to that of concussion. There is often insensibility, and, where immediate danger to life exists, there is always the characteristic depression of the heart's action. But intense heat of the sun, acting more gradually on a strong constitution not exhausted by fatigue, has often produced a state of insensibility, in which the pulse has been fuller than natural, and the vessels of the head turgid, and which has either been fatal in the way of coma, or has been relieved by copious evacuations, and the application of cold; and the same cause has often produced other diseased states connected with derangements of the cerebral circulation. In this case, the most injurious effect of the heat is evidently exerted on the vascular system exciting the heart, and probably expanding the blood in the vessels; and the brain suffers from increased compression, whereas in the former case the primary effect of the heat appears to be on the nervous system, the heart suffering from the violent impression made there.

III. It appears from experiments on animals, that lightning or other intense forms of electricity, likewise produce an effect similar to that of concussion, depressing or even extinguishing the action of the vascular system, and, at the same time, causing insensibility; but that the same agent, operating in a less intense degree produces insensibility without any immediate sedative effect on the circulation; that this state often appears to be connected with turgescence of the vessels of the head, and, probably, expansion of their contents; and that it may end in death by coma, or be followed by partial and more permanent injury of some of the nervous functions, or may be cured by depleting remedies.

IV. The effects of cold are very various according to circumstances, and depend not so much on the degree of cold that is applied to the body, nor even on the degree to which the body is actually cooled, as on the rapidity of the change, and probably, on the intensity of the sensation excited. It was found by Chossat that the temperature to which the bodies of animals killed by cold had been reduced before death varied considerably, and was always higher as the refrigeration had been more rapid, and therefore more injurious. The effect of cold on the living body has always been observed to be greater, as the sensation it excites is more intense and permanent, and therefore to be increased by all circumstances, either of the body exposed, or of the degree and mode of application of the cold, by which the intensity and duration of the sensation are increased. Cold, exerting its full effect on the body, is commonly stated to induce stupor, and death by coma. This effect, Dr. Alison remarks, has often been observed, and has sometimes been preceded by delirium, and attended by hemorrhage from the nostrils or ears, and has been found, on dissection, to be connected with considerable serous effusion in the head. It probably, therefore, depends in great measure on the flow of blood being much diminished to the surface and extremities, and proportionally increased to the brain. Persons recovering from this state of stupor by the assiduous application of heat, have continued comatose for hours after the circulation in the extremities has been restored. But in those who become comatose from cold, the heart's action is enfeebled, and it appears distinctly, from observation and experiment on man and animals, that the most intense cold may be fatal in the manner of a concussion; respiration continuing up to the moment when the heart's action ceases, the heart being found motionless, with arterial blood in its left cavities immediately after death, and artificial respiration being ineffectual in prolonging life. Cold always acts as a sedative on the capillary circulation on the surface of the body, and Dr. Edwards found that its repeated or long-continued application greatly diminished the power of subsequently generating heat. In some cases of frostbite, this effect is so powerful on the parts implicated as to put an entire stop to their vital action, even when the general system does not materially suffer; and the sedative effect on the vital actions of the frostbitten part is frequently such that the inflammation excited in a greater or less degree by the return of heat, is characterized by deficient reaction, and tends rapidly to gangrene. But these effects of cold on the vital actions of *individual parts* must be carefully distinguished from its powerful sedative operation on the *whole system;* because, in the first case, when the general circulation is strong, the chief danger is from the inflammation which ensues on the reestablishment of the circulation and natural heat of the part, and is to be moderated by causing their restoration to take place very gradually; whereas, when the vital power of the whole system has been depressed, there is no such risk of local injury from an elevation of temperature and external heat, and other stimuli may be more freely applied.

V. In regard to the action of *poisons,* Dr. Alison adverts to the following questions:

1. Is it essential to their action that they should be absorbed into the circulation, or may they act on the nerves of the parts to which they are directly applied, and affect sympathetically the organs more essential to life? This much-disputed question he decides—we think justly—in favour of both modes of operation, but concludes that the greater part of the effect of poisons which have been absorbed into the blood, is consequent on their direct application to the more important vital organs. He adds, that while the action of some poisons taken into the blood has been observed to be attended by a change in the sensible qualities of that fluid, and especially by diminution or loss of its coagulability, many other poisons act fully without altering its sensible properties, so that, where such alteration occurs, it is doubtful how far it may be connected with the action of the poisons on the living solids; the loss of coagulability in the blood also is so common in cases of sudden death, that of itself it throws no light on the immediate cause of the fatal event. It is important to keep these considerations in mind when reasoning analogically from poisons to malignant diseases, since they show that, although the blood be changed by the agency of the cause of these diseases, it does not necessarily follow that such change is concerned in producing the most essential symptoms, or the fatal termination.

2. When a poison has been absorbed into the blood, is its action on the circulation itself to be ascribed to its direct contact with the heart and vessels, or, in a great measure, to an influence transmitted to them from the nervous system, which the poison must also necessarily pervade?

"On this point," says Dr. Alison, "all that can be stated is, that we have clear evidence of the noxious effect of many poisons on moving solids to which they are directly applied (*e. g.* on those of the fibres of the heart or intestines with which they are laid in contact), or even on vegetables; but, nevertheless, as we have seen that the action of all parts of the circulating system in animals is subjected to an influence or control, from changes taking place in the nervous system, it is quite possible that the agency of poisons circulating in the blood on muscular organs, and especially on the circulation, may be in part consequent on the impression which they make on the brain and nerves. And the *order of the symptoms* in the case of some such poisons—as the oxalic acid—would seem to denote that the primary effect is on the brain and spinal cord, and that the heart suffers secondarily." (p. 21.)

3. The action of the different mortal poisons clearly exemplifies two of the modes of sudden death, namely, death by coma and death by syncope. Some poisons appear to have a peculiar action on the lungs, but the only case in which they act strictly by asphyxia is that where certain gases—as carbonic acid in large quantity—excite violent spasm at the glottis.

*a.* The narcotic poisons produce *coma*, and the immediate cause of death consequent on their action is arrest of the pulmonary circulation; but this ultimate effect, as in the instance of injuries of the brain, is preceded by various affections of the nervous system, as delirium, convulsions, vertigo, loss of different external senses, &c., or it may take place gradually, without any of these. *b.* Other poisons are evidently fatal by *syncope*, the unequivocal indications of which kind of death are, that the respiration continues as long as the action of the heart, and that the heart is therefore found, immediately after death, motionless, unexcitable by stimuli, and filled with venous blood on the right side and arterial on the left. It is thus that death is produced by the upas antiar, and by infusion of tobacco; other poisons, as hydrocyanic acid, digitalis, strychnine, oxalic acid, arsenic, preparations of antimony and baryta, various animal poisons, &c., appear, in full doses, to act chiefly by syncope, though they have also a more complex operation. *c.* Some vegetable and many mineral poisons excite inflammation of the gastro-enteric mucous membrane, or other textures, and the symptoms of such phlegmasiæ complicate those of the direct agency of the poisons on the nervous or vascular system, and are sometimes the principal cause of death. *d.* Some poisons, taken into the system, operate slowly, and their effects are regarded simply as diseases, sometimes not to be distinguished from others which may be excited by ordinary causes, as when arsenic produces epilepsy—lead, colic and palsy, &c.

VI. In a general way the modus operandi of fatal hemorrhage is too obvious to demand notice here: the following remark, however, is worthy of attention:

"A more sudden and violent hemorrhage affects the nervous system much more speedily—just as we have already seen—than any other means of suddenly diminishing the pressure to which the brain had been subjected does; and the impression thus made in the brain *reacts on the heart after the manner of a concussion,* and causes its action to fail much sooner than it would have done merely by reason of the loss of blood. It is only in this way that we can explain the fact, that in bleeding from a large orifice, and in the erect posture, not only sensation and the other functions of the brain are sooner suspended, but *the heart's own actions fail,* with much less loss of blood than when the orifice is smaller, and the patient lies horizontally, so that the diminution of the pressure on the brain is less and more gradual." (pp. 26-7.)

Death (VII) by *fasting,* and (VIII) by *asphyxia,* need not detain us, as our author's remarks on them are brief, and illustrate no particular analogy that has not been already adverted to.

We wish that our space permitted us to present the reader with such an analysis of the three following chapters as we have made of the first. These chapters are on "Diseases in general—their fatal terminations, or spontaneous decline;" on "The remote causes of diseases in general, and the means of their prevention;" and on "The action of remedies in general, and the evidence of their efficacy." They constitute the first part of the work before us; and to this, as exhibiting a bold and masterly outline of the philosophy of practical medicine, we wish particularly to draw the attention of our readers.

Part II treats of "Febrile diseases," including, under that denomination, the various phlegmasiæ, idiopathic fevers, and the contagious exanthemata. In the first chapter the author enters at great length into the subject of inflammation in general—a subject which we have treated so fully of elsewhere, that it is unnecessary to enter upon it here. We cannot, however, avoid observing that Dr. Alison's theoretical views of inflammation, while they exhibit much of the close and subtle reasoning characteristic of his writings, present here and there instances of positive conclusions arrived at on what appears to us very insufficient grounds.

Part III embraces the consideration of "Chronic or non-febrile diseases." It is of equal excellence with the two preceding, and, like them, combines the philosophy with the practice of medicine in a manner surpassed by no work but Cullen's 'First Lines.' In the concluding chapter, on "Chronic diseases of the nervous system and organs of sense," the student will find a very valuable guide to the proper method of studying that obscure but highly interesting department of practical medicine. In conclusion, we beg to recommend Dr. Alison's work in the strongest terms to every medical student and to the whole profession. The subtlest reasoner on the science, and the simplest practitioner or student in the art of medicine, will each find in it materials admirably suited to his purpose. It is, in truth, no ordinary book, and the production of no ordinary man.

